# Acquisition and annotation in high resolution *in vivo* digital biopsy by confocal microscopy for diagnosis in oral precancer and cancer

**DOI:** 10.3389/fonc.2023.1209261

**Published:** 2023-07-03

**Authors:** Tami Yap, Ivy Tan, Rishi S. Ramani, Nirav Bhatia, Paula Demetrio de Souza Franca, Chris Angel, Caroline Moore, Thomas Reiner, Lindsay Bussau, Michael J. McCullough

**Affiliations:** ^1^ Melbourne Dental School, Faculty of Medicine, Dentistry and Health Sciences, Carlton, VIC, Australia; ^2^ Oral Medicine Unit, Royal Dental Hospital of Melbourne, Carlton, VIC, Australia; ^3^ Department of Radiology, Memorial Sloan Kettering Cancer Center, New York, NY, United States; ^4^ Department of Otorhinolaryngology and Head and Neck Surgery, Federal University of São Paulo, São Paulo, SP, Brazil; ^5^ Department of Pathology, Peter MacCallum Cancer Centre, Victorian Comprehensive Cancer Centre, Parkville, VIC, Australia; ^6^ Optiscan Imaging, Mulgrave, VIC, Australia

**Keywords:** oral cancer, confocal, endomicroscope, OSCC (oral squamous cell carcinoma), dysplasia, fluorescence, oral potentially malignancy disorders (OPMDs), digital health

## Abstract

**Introduction:**

Scanned fibre endomicroscopes are full point-scanning confocal microscopes with submicron lateral resolution with an optical slice thickness thin enough to isolate individual cell layers, allow active positioning of the optical slice in the z-axis and collection of megapixel images. Here we present descriptive findings and a brief atlas of an acquisition and annotation protocol high resolution *in vivo* capture of oral mucosal pathology including oral squamous cell carcinoma and dysplasia using a fluorescence scanned fibre endomicroscope with 3 topical fluorescent imaging agents: fluorescein, acriflavine and PARPi-FL.

**Methods:**

Digital biopsy was successfully performed *via* an acquisition protocol in seventy-one patients presenting for investigation of oral mucosal abnormalities using a miniaturized, handheld scanned fibre endoscope. Multiple imaging agents were utilized and multiple time points sampled. Fifty-nine patients had a matched histopathology correlating in location with imaging. The images were annotated back to macrographic location using a purpose-built software, MouthMap™.

**Results:**

Acquisition and annotation of cellular level resolved images was demonstrated with all 3 topical agents. Descriptive observations between clinically or histologically normal oral mucosa showed regular intranuclear distance, a regular nuclear profile and fluorescent homogeneity. This was dependent on the intraoral location and type of epithelium being observed. Key features of malignancy were a loss of intranuclear distance, disordered nuclear clustering and irregular nuclear fluorescence intensity and size. Perinuclear fluorescent granules were seen in the absence of irregular nuclear features in lichenoid inflammation.

**Discussion:**

High resolution oral biopsy allows for painless and rapid capture of multiple mucosal sites, resulting in more data points to increase diagnostic precision. High resolution digital micrographs can be easily compared serially across multiple time points utilizing an annotation software. In the present study we have demonstrated realization of a high-resolution digital biopsy protocol of the oral mucosa for utility in the diagnosis of oral cancer and precancer..

## 1 Introduction

Despite the current global trend in tobacco reduction, oral squamous cell carcinoma (OSCC) is still an important health concern. Individuals diagnosed at advanced stages undergo extensive surgery and postoperative adjuvant therapies with resultant impact on quality of life. Furthermore, patients with late stage OSCC experience a poorer 5-year disease-survival rate of 40.7%. However, with early stage diagnosis, prognosis has a much higher five-year survival rate, reaching over 84% (1). Therefore, timely detection plays a crucial role in improving the survival rates and post-treatment quality of life for patients with OSCC.

The diagnosis of OSCC at an early stage requires using a combination of conventional expert oral examination and scalpel biopsy (2). Oral potentially malignant disorders (OPMDS) are visible changes of the oral mucosa that are associated with transformation into and increased risk of OSCC that affect up to 5% of the general population (3). The most common OPMDs are oral leukoplakia, oral lichen planus (OLP), and oral lichenoid lesions (OLL) with an overall malignant transformation rate varying from 1.1 to 4.3% (4, 5). Although screening for OPMDS increases the chance of early stage diagnosis (6), there currently remains limited capacity for prediction of the development of malignancy in any one individual and for any particular oral lesion. Diagnostic challenges include the lack of uniform progression rates (ranging from 6%–36%) and variable association with degrees of dysplasia in OPMDs.

Invasive intraoral biopsies are typically performed under local anesthetic in an outpatient setting. These procedures cause anxiety in patients, resulting in subsequent oral post-procedural pain. Further, histopathological conclusions made on the tissue sample excised are presumptively applied to the remainder of abnormal mucosa. Non-invasive tools that aim to provide cellular level resolved, rapid, multisite insight beyond white light visualization of the oral mucosa is being continually developed.

Here we use high-resolution fluorescence confocal microscopy with 3 different topical fluorescence agents to address this clinical need. Confocal microscopy is an optical imaging technique that increases optical resolution and contrast of a micrograph by means of adding a spatial pinhole placed at the confocal plane of the lens to eliminate out-of-focus light (7). It allows for real-time, non-invasive, microscopic field of view assessment of material of interest.

Confocal microscopes may utilize reflectance or fluorescence imaging, or both (8). The structural visualization of reflectance confocal microscopy is dependent on the refractive index differences in the tissue and doesn’t require introduction of a fluorophore into the tissue. Thereby, it is not limited by fluorescence latency (9). Reflectance confocal microscopy has been explored in diagnosis of oral mucosal disease including oral dysplasia, oral lichen planus and cancer extensively by Contaldo et al. (10, 11) Fluorescence confocal microscopy requires introduction of a fluorophore into the tissue that allows for targeting of specific structures, which may preferentially bind the fluorophore allowing for targeted visualization. However, miniaturization of capturing probe to be introduced in the oral cavity is an important consideration when using confocal microscopes in assessing oral mucosal disease. Another acquisition consideration is the mobility of *in vivo* oral tissues and the presence of saliva in a patient who is seen in an outpatient setting without sedation or general anesthesia.

Highly miniaturized handheld confocal microscopes for use *in vivo* have been developed, and these are known as endomicroscopes. There are 2 types of endomicroscope - scanned fibre and bundle fibre devices. The most important difference between the 2 technologies in the fundamental context of cancer diagnosis is resolution. The imaging resolution of bundle fibre devices is limited by the number of fibres in the bundle, the fixed z-focus-depth and the thick z-section collected by the device. Only one focal point per fiber can be collected, thus limiting the resolution. Typically, bundle fibre devices cannot resolve cells and subcellular features required for diagnostic assessment (12). Differently, scanned fibre endomicroscopes are full point-scanning confocal microscopes with submicron lateral resolution and an optical slice thickness thin enough to isolate individual cell layers, active positioning of the optical slice in the z-axis and collection of megapixel images. What this means is that scanned fibre endomicroscopes collect images with cellular and subcellular resolution enabling assessment of histological features in real-time *in vivo* images. Scanned fibre confocal microscopy has been used for *in vivo* identification of cancer, precancer and other diseases of the mouth, esophagus, cervix, colon and brain (13–19).

Employed topical fluorescent contrast dyes used for epithelial architecture visualization in mucosal studies includes acriflavine, a common topical antiseptic, and fluorescein, an exogenous dye used routinely in ophthalmic practice for detection of foreign bodies and corneal abrasions (20, 21). Fluorescein and acriflavine can be considered pancytoarchiteral fluorescent agents as they non-selectively bind membrane and nuclear material, regardless of molecular activity. Acriflavine has been used in combination with several types of confocal microscope technology, including point scanning to visualize and diagnose oral mucosal disease (18, 22). PARP1 (Poly (ADP-ribose) polymerase 1 is a nuclear protein that has been found to be overexpressed in many malignancies including ovarian cancer, breast cancer and OSCC (23). Reiner et al. first reported the small molecule PARPi-FL, a fluorescent dye-based imaging agent, that binds to PARP1 (24). PARPi-FL is based on the PARP1 inhibitor olaparib (Lynparza) that is FDA-approved for the treatment of several types of cancer, but mostly used for the treatment of ovarian cancer. PARPi-FL is intentioned as an indicator of PARP1 activity and has been shown to delineate tumor detection from normal tissue in mice models, *ex vivo* human tissue (25), and *in vivo* in a Phase I clinical trial as a mouthwash (26). PARPi-FL can be used as a molecule-specific fluorescence agent.

Another important consideration with cellular level imaging is reference back to the mucosal site from which the image was captured. Further, to allow multiple time points or locations to be easily and practically compared, an annotation tool to map the mouth is needed.

The aims of the present study were to

- validate the use of a scanned fibre endomicroscope protocol to perform non-invasive, real-time high resolution digital biopsy of the oral mucosa.
- present a brief image atlas that documents the comparative features of *in vivo* microscopy of histopathology-diagnosed oral mucosal conditions.
- demonstrate the use of 3 different topical fluorophores in acquiring cellular level resolved images.

## 2 Material and methods

This exploratory, phase I, single-center, open-label, prospective Privacy Act of 1988 compliant study was approved by the University of Melbourne Medicine and Dentistry Human Ethics Sub-Committee (ID 1955205) and conducted in accordance with the Declaration of Helsinki. Written informed consent was obtained from all patients.

### 2.1 Participants

Patients were consecutively recruited from the Oral Medicine Department of the Royal Dental Hospital Melbourne. Individuals attending for assessment of oral mucosal abnormalities, including OPMDs or with a history of oral cancer were invited to participate.

### 2.2 Synthesis of PARPi-FL

PARPi-FL was produced by our collaborators at Memorial Sloan Kettering (MSK) Cancer Center in New York, USA, as previously described (27), and fast shipped to Australia in dry ice. Briefly, **t**riethylamine (NEt_3_) >99.5% purity, Trifluoroacetic acid (TFA) ReagentPlus 99%, and dimethyl sulfoxide-d_6_ (DMSO-d_6_) were purchased from Sigma-Aldrich (St. Louis, MO). Anhydrous acetonitrile (CH_3_CN) over molecular sieve, HPLC-grade acetonitrile, BODIPY FL NHS ester, and Texwipe Technicloth were purchased from Thermo Fisher Scientific (Waltham, MA). Water (18.2 MΩ cm^-1^ at 25 °C) was obtained from an Alpha-Q Ultrapure water filtration system from Millipore (Bedford, MA). PARP-NH precursor (4-(4-fluoro-3-(piperazine-1-carbonyl)benzyl)phthalazin-1(2H)-one) was synthesized at MSK according to protocols reported earlier. PARPi-FL was produced under good manufacturing practice (GMP) conditions at MSK under IND number 133,109. Briefly, BODIPY-FL NHS ester (5.0 mg, 12.8 μmol, 1.0 eq.) was conjugated to 4-(4-fluoro-3-(piperazine-1-carbonyl) benzyl) phthalazin-1(2H)-one (9.4 mg, 25.6 μmol, 2.0 eq.) in the presence of Et_3_N (6.3 μL) in anhydrous acetonitrile for 4 h at room temperature. Purification by preparative HPLC (Phenomenex Jupiter 5u C18 300A, 10 × 250 mm, 3 mL/min, 5 to 95% of acetonitrile (0.1% TFA) in 15 min) and subsequent lyophilization yielded PARPi-FL (5.9 mg, 73%) as a red solid. Analytical HPLC analysis (Waters’ Atlantis T3 C18 5 μm, 4.6 × 250 mm, 3 mL/min, 5 to 95% of acetonitrile (0.1% TFA) in 15 min) showed high purity (99.27%, *t_R_* = 14.2 min) of the imaging agent. The identity of PARPi-FL was confirmed using LC-MS (MS (+) *m/z* = 621.15 [M-F]^+^) and NMR (consistent with structure).

### 2.3 Confocal microscope

**Figure 1:**
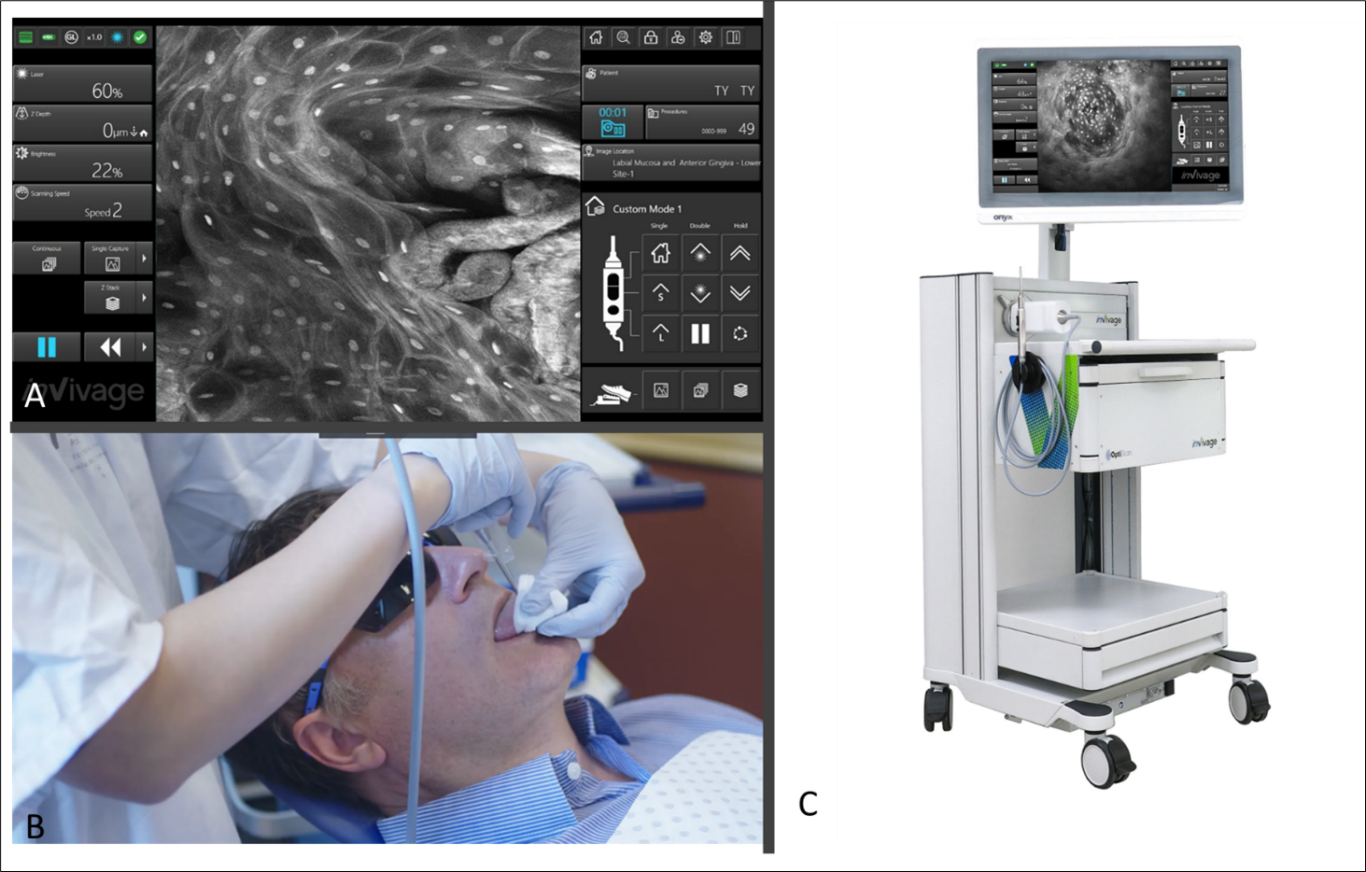
**(A)** Software interface of the InVivage® demonstrating laser and capture settings on the left-hand side and location and patient selection on the right-hand side panel **(B)** Handheld intraoral probe and command handpiece allows the user to adjust settings without de-gloving **(C)** The portable InVivage® unit can be transported alongside a conventional dental chair.

The InVivage®(Optiscan Imaging, Victoria Australia) system at the time of this study was an investigational medical device for the purposes of this study only. The system has a software interface which allows for adjustment of laser power, depth, refresh rate, capture methods and macro data which is connected to the image output which can be selected to map back based on the chosen intraoral location (**Figure 1**). Briefly, the InVivage® system is a hand-held scanned fibre confocal endomicroscope that acquires images using a single channel for illumination and detection of 488 nm excitation, with a lens NA of 0.3 (similar to a 10 × objective) and a field of view of 475 µm × 475 µm. The resolution capabilities are 0.55 µm lateral and 5.1 µm axial The system is used by placing the probe in direct contact with the tissue, after treatment with a dye, to acquire images at different depths along the Z axis (28). The physical image plane can be translated from the surface to 400 µm but imaging depth with topical application was typically from tissue surface to a depth of 100 µm. Further setting details are described below under Image Acquisition.

### 2.4 Clinical protocol

The clinical protocol was as follows:

1) Oral cavity examination performed by an oral medicine specialist that included a systematic inspection of all mucosal surfaces.
2) Registration of all intraoral lesions by macrographic photography (Canon EOS 200D Canon 100mm F2.8 Macro USM)
3) Confocal microscope imaging (InVivage®). The patients were imaged with a single or multiple agents in the following order:
a. *PARPi-FL* Patients were instructed to swish a 10 mL solution of 1µM PARPi-FL in 30% PEG 300 and 70% water for 1 minute, followed by a 30-second water rinse, and then undergo digital microscopic fluorescence imaging.
b. *Fluorescein* - Patients were asked to swish 10 mL of a 0.1% solution of fluorescein in sterile water for 1 minute, followed by a water rinse that was repeated until the rinsed water appeared clear. Then, they underwent digital microscopic fluorescence imaging.
c. *Acriflavine* – Mucosal areas of interest were painted with a cotton swab soaked in a solution of 0.1% acriflavine in sterile water, followed by a 1-minute water rinse that was repeated until the rinsed water appeared clear. Digital microscopic fluorescence imaging was then performed.

### 2.5 Image acquisition

**Figure 2:**
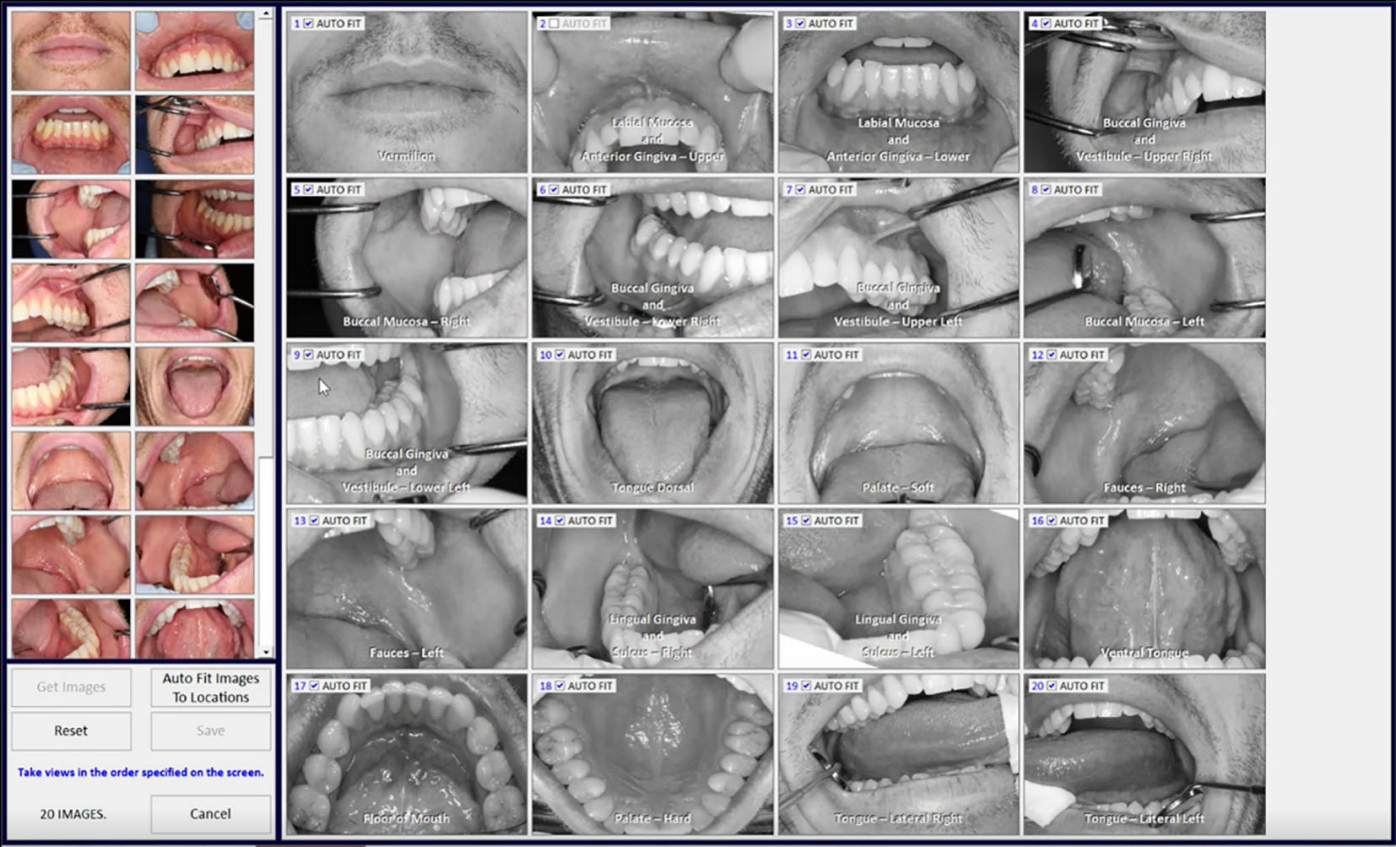
MouthMap™ interface showing macrographs allocated into the 20 intraoral locations.

**Figure 3:**
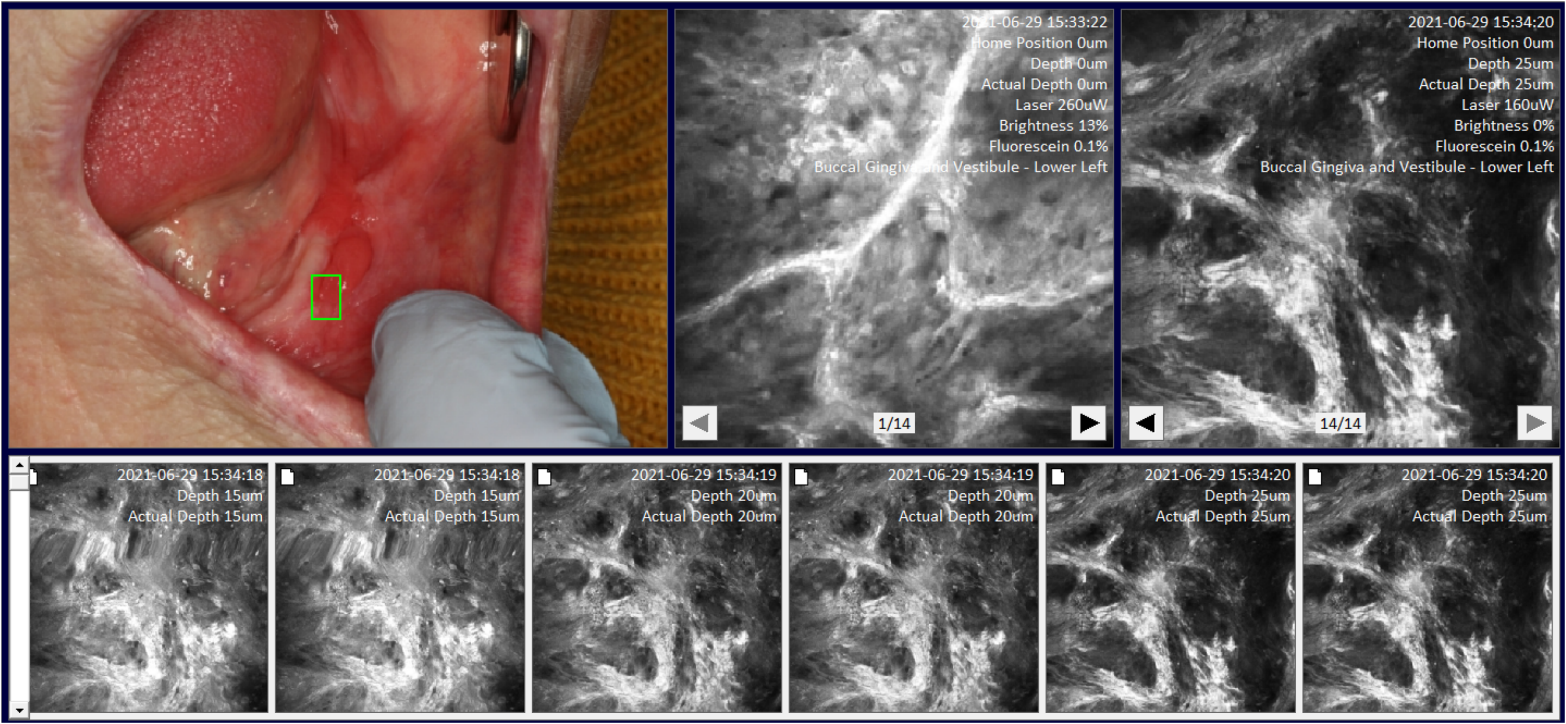
Site specific micrographs of the right lateral surface of the tongue visualized in MouthMap™ demonstrating time stamp, location, and laser setting image macros. Image captures are 475µm (W) x 475µm (H).

Image location-site selection was dependent on the intraoral location of mucosal abnormality. The terms are defined as follows: image location was defined as one of 20 intraoral areas which together captured all areas of the oral cavity by macrographic photography and was compatible with the mapping software we developed called MouthMap™ (**Figures 2**, **3**.). Image site was defined as the specific point at which the probe was placed when the image capture occurred. An image set was defined as a set of images acquired using a single fluorescence agent collated by location and sites.

The control location-sites were completed at the buccal mucosa and/or the contralateral surface if clinically normal oral mucosa was present. Capture at an image site was made by placing the probe on the site of interest and capturing a Z-stack. The Z-stack image acquisition commences at the surface of the probe into the tissue until the depth of 100µm with 5 µm incremental focal plane advancements. This Z-stack command occurred utilizing a programmed single foot pedal command (**Figure 2**) so that the user could remain otherwise immobile whilst supporting the intraoral tissues. For all patient imaging, we used long pass filter of 515– 815 nm, and a scanning speed of 1024 pixels x 512 lines with a frame rate of 0.7 seconds per frame. The settings of the laser power are set at 100%, when imaging with PARPi-FL, and between 10-25% when imaging with fluorescein and acriflavine.

### 2.6 Location annotation

We developed a custom-made software, MouthMap™ which was used to map micrographs captured with the InVivage® and paired with software-housed macrographs classified under 20 intraoral locations (**Figure 2**). Location and site linkage used the macro data captured on the micrograph Dicom files which link back to the macroscopic intraoral location. Sites were annotated on the macrographs and the micrographic images were linked and uploaded for visualization (**Figure 3**).

### 2.7 Diagnosis

Standard of care scalpel biopsy and diagnosis by histopathology was completed at the discretion of the attending clinician. Indication for prospective biopsy included if histopathology was required for definitive diagnosis of a new or changed clinical presentation. Selection of biopsy site was selected as per standard care and before applying the confocal microscope.

### 2.8 Longitudinal capture

Participants were reappointed for review of their oral lesions as per standard of care. During the review periods, these individuals may have undergone medical therapy, excision of their lesions or regular monitoring.

### 2.9 Data management

Clinical data was collected under a deidentified research ID and managed using REDCap electronic data capture tools hosted at the University of Melbourne (29, 30).

## 3 Results

### 3.1 Patient cohort

Seventy-one patients were recruited for this project, including 33 women and 38 men (43.5% and 53.5% respectively) with a mean age of 64 ± 12.2 years old (range 32 - 88 years). Patients were seen and imaged 1 to 5 times during the study period. Fifty-four (76.0%) of patients presented with a keratotic and/or erythematous mucosal abnormality of the oral cavity mucosa. Histological correlation was obtained from 83% (n = 59) of the imaged patients (**Table 1**). Histopathological diagnostic categories included hyperkeratosis or hyperplasia, no dysplasia, dysplasia, oral lichenoid inflammation, or oral squamous cell carcinoma. Out of the 12 patients that did not have a histopathological correlation, 8 patients’ cases being followed up for oral lichen planus or oral lichenoid lesions diagnosed with a previous biopsy (prior to the inclusion in this protocol) and 4 patients had a clinical diagnosis of either benign alveolar ridge keratosis (n=2), post-excision scar (n=1) or traumatic ulceration (n= 1).

**Table 1:** Histopathological conclusion of 59 micrograph matched biopsies.

Histopathological Conclusion	(n of 59)
squamous cell carcinoma	3
high grade dysplasia	5
low grade dysplasia	7
atypia insufficient for dysplasia	2
verrucous hyperplasia	2
hyperkeratosis and or hyperplasia	17
consistent with OLP or lichenoid inflammation	13
Other:	
amalgam tattoo	1
chronic inflammation	2
denture associated gingival hyperplasia	2
fibroepithelial polyp	2
papilloma or focal papillomatosis	2
verruciform xanthoma	1

### 3.2 Types of imaging agents

**Figure 4:**
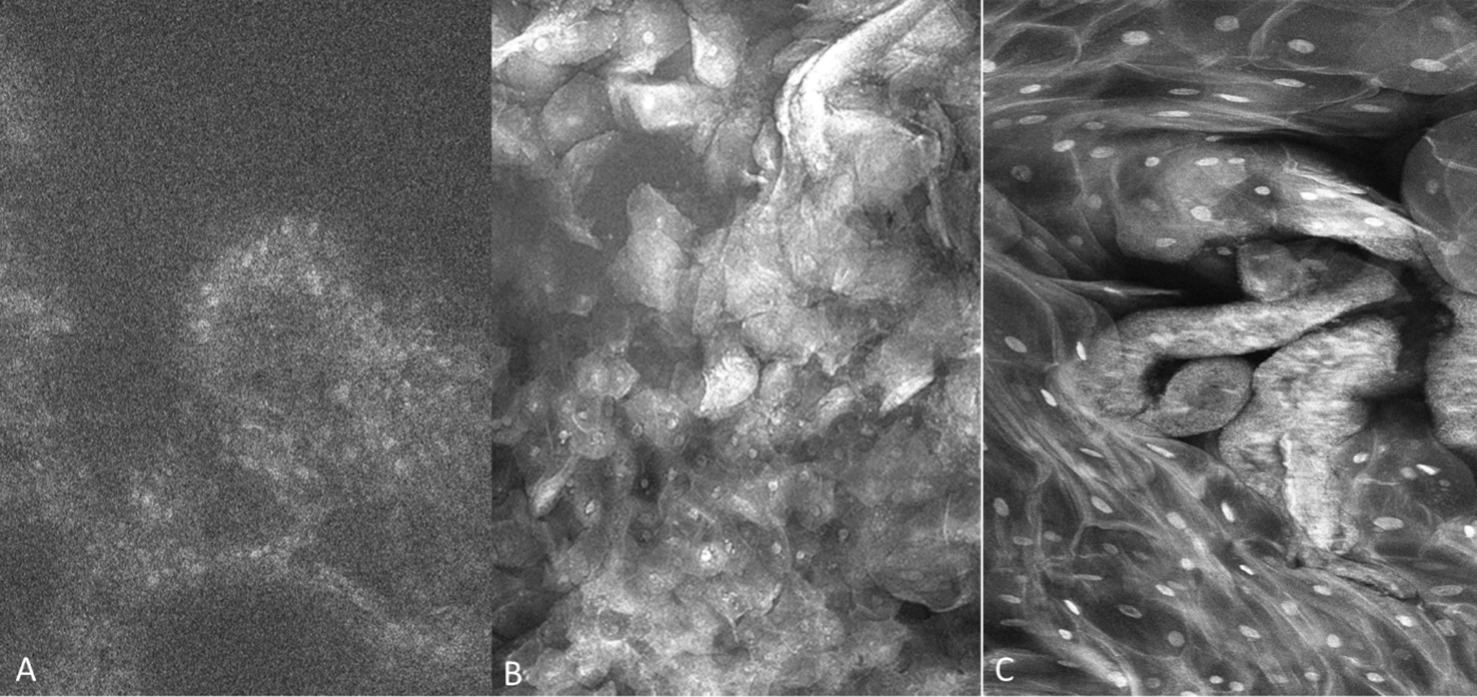
Fluorescence images capture with molecule-targeted 1µM topical PARPi-FL **(A)** and pan-cytoarchitectural fluorescent agents topical 0.1% fluorescein **(B)** and 0.1% acriflavine **(C)**. Images are 335µm (W) x 475µm (H).

A total of 307 imaging sets were captured which included 109 using PARPi-FL, 93 using fluorescein and 105 using acriflavine. Successful imaging acquisition with cellular level resolution was achieved with all 3 imaging agents: PARPi-FL, acriflavine and fluorescein using the described protocol. PARPi-FL was utilized as a molecule-targeted fluorescence agent. It was expected to bind to less nuclear material than the pancytoarchitectural dyes, and therefore have less signal flooding. The majority of imaged lesions did not have nuclear PARPi-FL pickup. Some non-specific, non-nuclear PARPi-FL signal was observed in highly keratinized lesions similar to that found with acriflavine and fluorescein. The fluorescein stain provided better visualization of cell membranes, whereas the acriflavine stain provided better nuclear visualization (**Figures 4A–C**).

### 3.3 Intraoral regional mucosal variation

**Figure 5:**
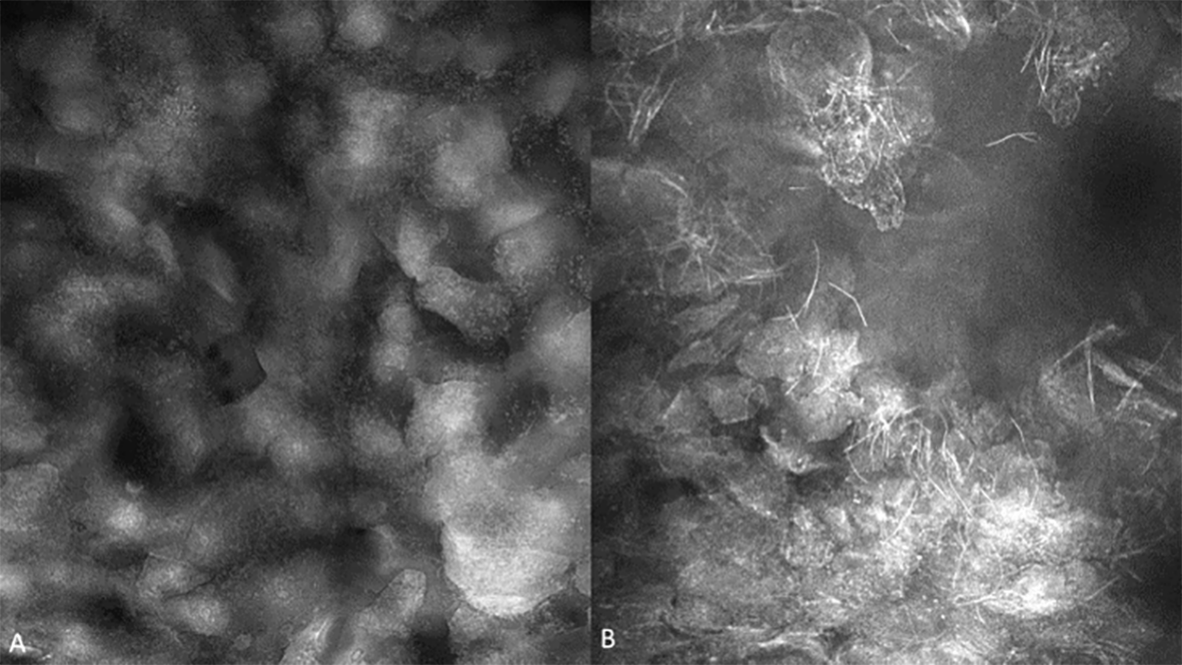
**(A)** Bacterial plaque and pellicle debris visualized on the gingiva; **(B)** Candidal hyphae observed in verrucous hyperplasia of the buccal mucosa with 0.1% fluorescein. Images are 422 µm (W) x 400µm (H).

**Figure 6:**
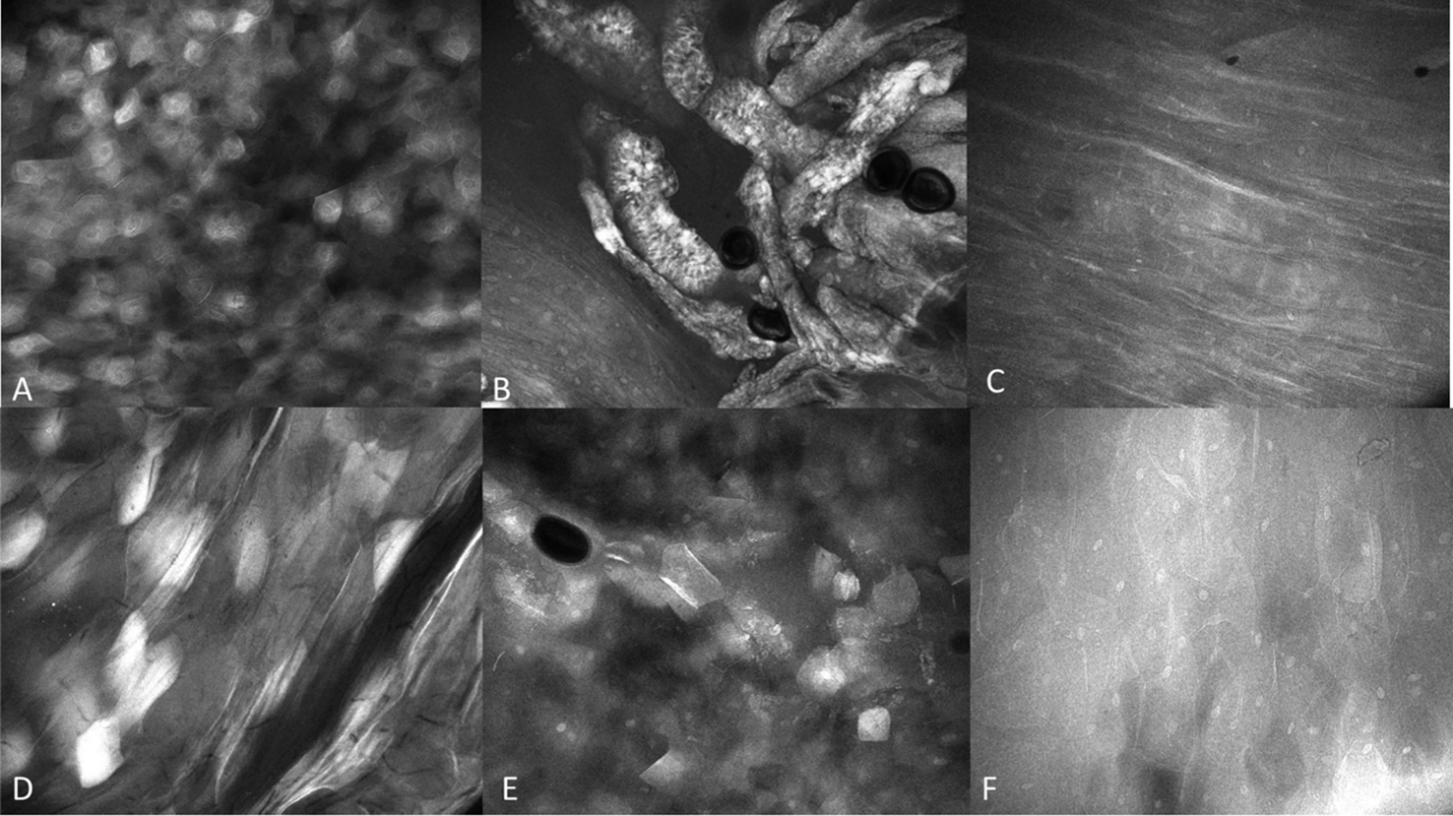
Regional mucosal variation with the InVivage® utilizing topical 0.1% fluorescein **(A)** keratinized epithelium of the hard palate, **(B)** specialized epithelium with papillae of the dorsal tongue, **(C)** transitional epithelium of the lateral tongue, **(D)** non-keratinized epithelium of the soft palate, **(E)** gingival epithelium, **(F)** buccal mucosa with fluorescein. Images are 475µm (W) x 400µm (H).

Observations of clinically normal oral mucosa show generally regular intranuclear distance between the keratinocyte cells, a regular cellular outline, and a regular nuclear profile. A typical regular nuclear profile consists of homogenous nuclear fluorescence and size. However, there was an observable microscopic variation in the visualization of clinically normal oral mucosa. This was dependent on the location and type of epithelium being observed. These variations differed depending on if the mucosa is keratinized vs non keratinized, or in an area where a microbial plaque was expected, such as the gingiva (**Figure 5**). Microbial presence was also observed (**Figure 6**).

### 3.4 Oral squamous cell carcinoma

**Figure 7:**
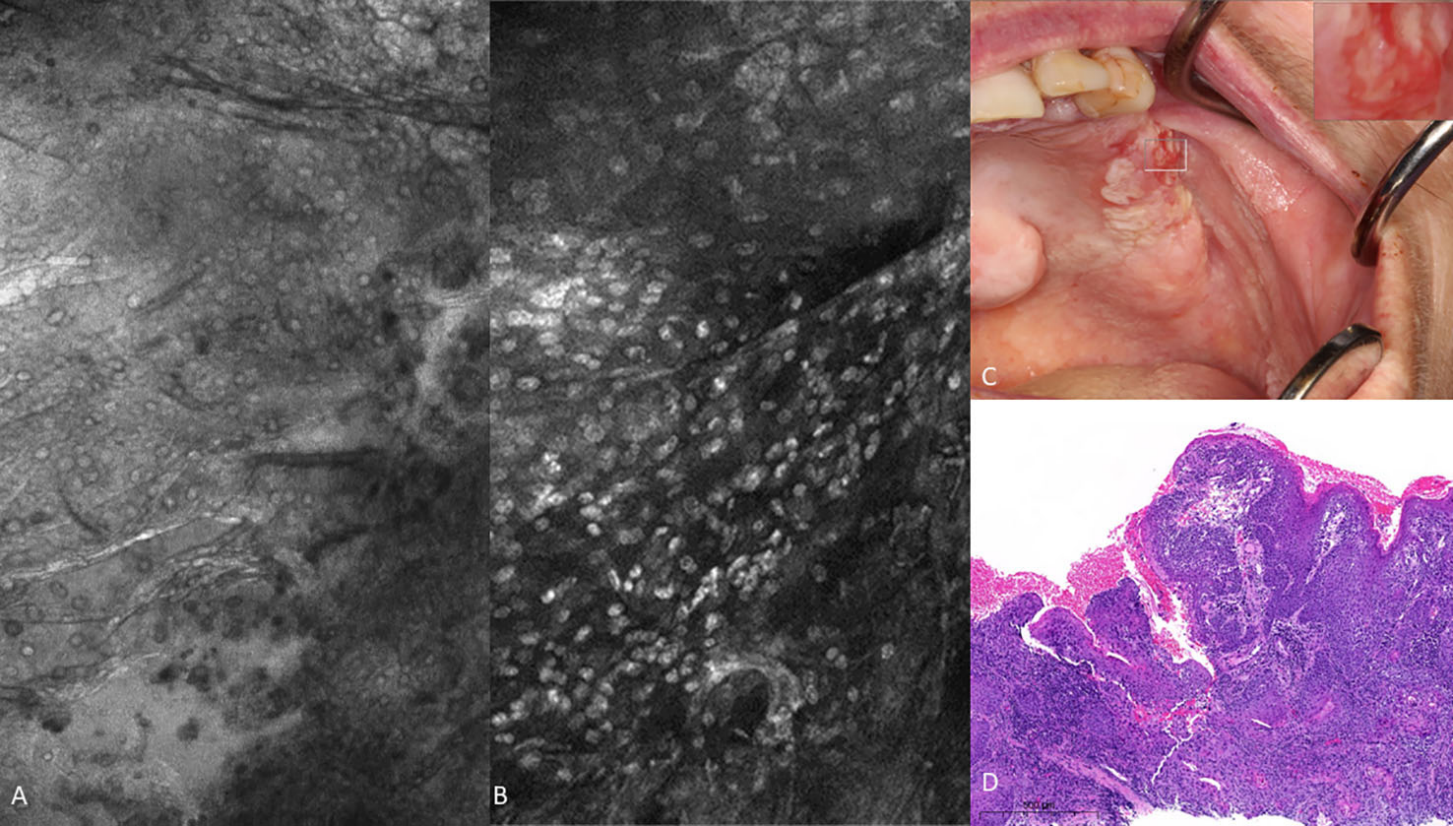
Oral squamous cell carcinoma of the left edentulous maxillary alveolus **(A)** Micrograph using topical 0.1% fluorescein [282µm (W) x 475µm (H)], **(B)** Micrograph using 0.1% acriflavine [282µm (W) x 475µm (H)], **(C)** Digital macrograph of red and white lesion on left maxillary alveolus, inset upper right corner is enlargement of area using magnifying visual tool in MouthMap ™ **(D)** H&E-stained histopathology (500µms scale bar).

**Figure 8:**
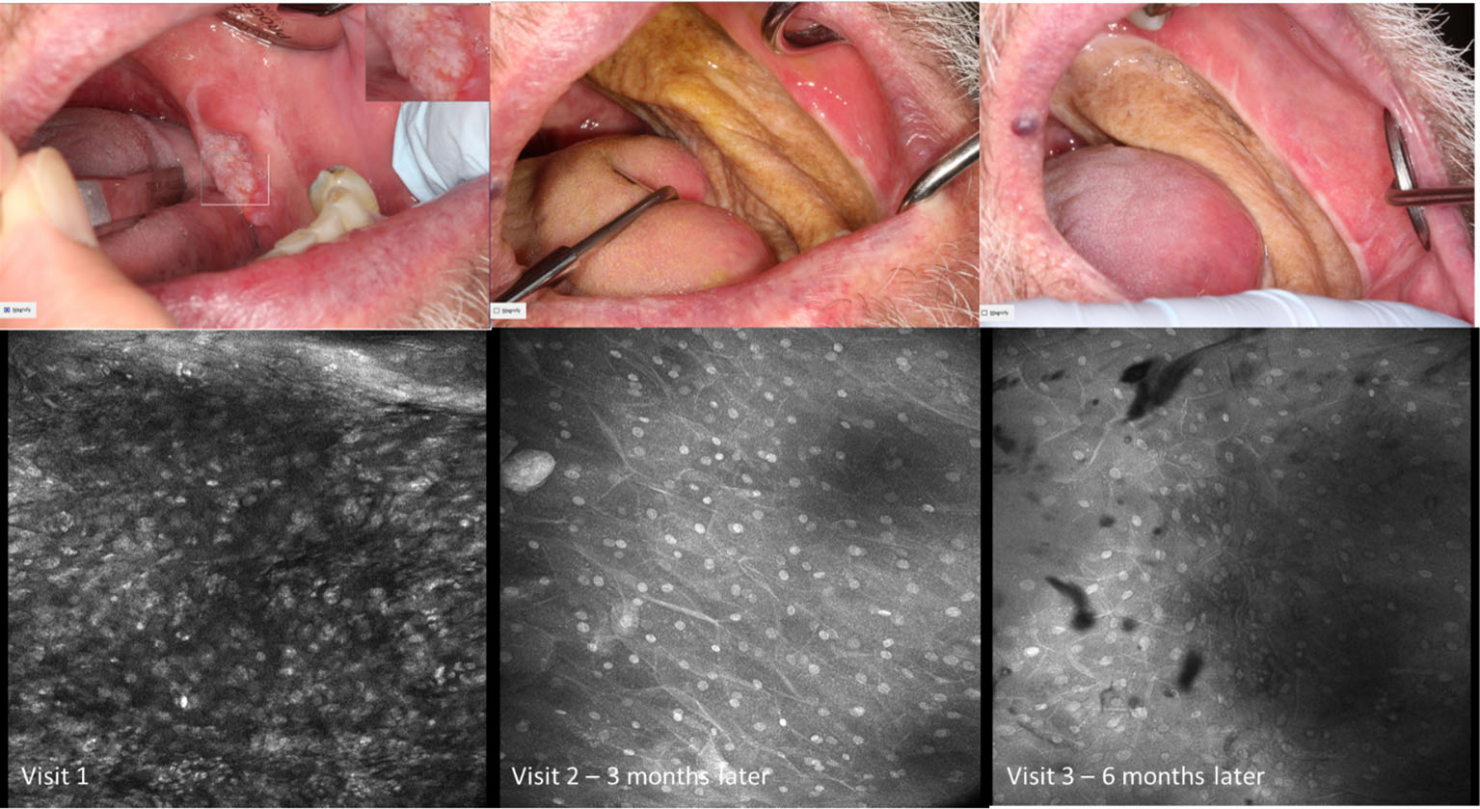
Micrography over time with topical 0.1% acriflavine demonstrating oral squamous cell carcinoma at Visit 1 and subsequent clinically normal non-keratinized mucosa adjacent to radial forearm free flap following resection. Images are 475µm (W) x 475µm (H).

There were obvious and striking visual differences between micrographs captured of normal mucosa and OSCC (**Figures 7**, **8**). Key features in micrographs demonstrating malignancy with both fluorescein and acriflavine were a loss of intranuclear distance, disordered nuclear clustering and irregular nuclear fluorescence intensity and size. There was a loss of cellular outline and definition, with the nuclei appearing globular, crowded and or clumped visually.

### 3.5 Oral dysplasia

**Figure 9:**
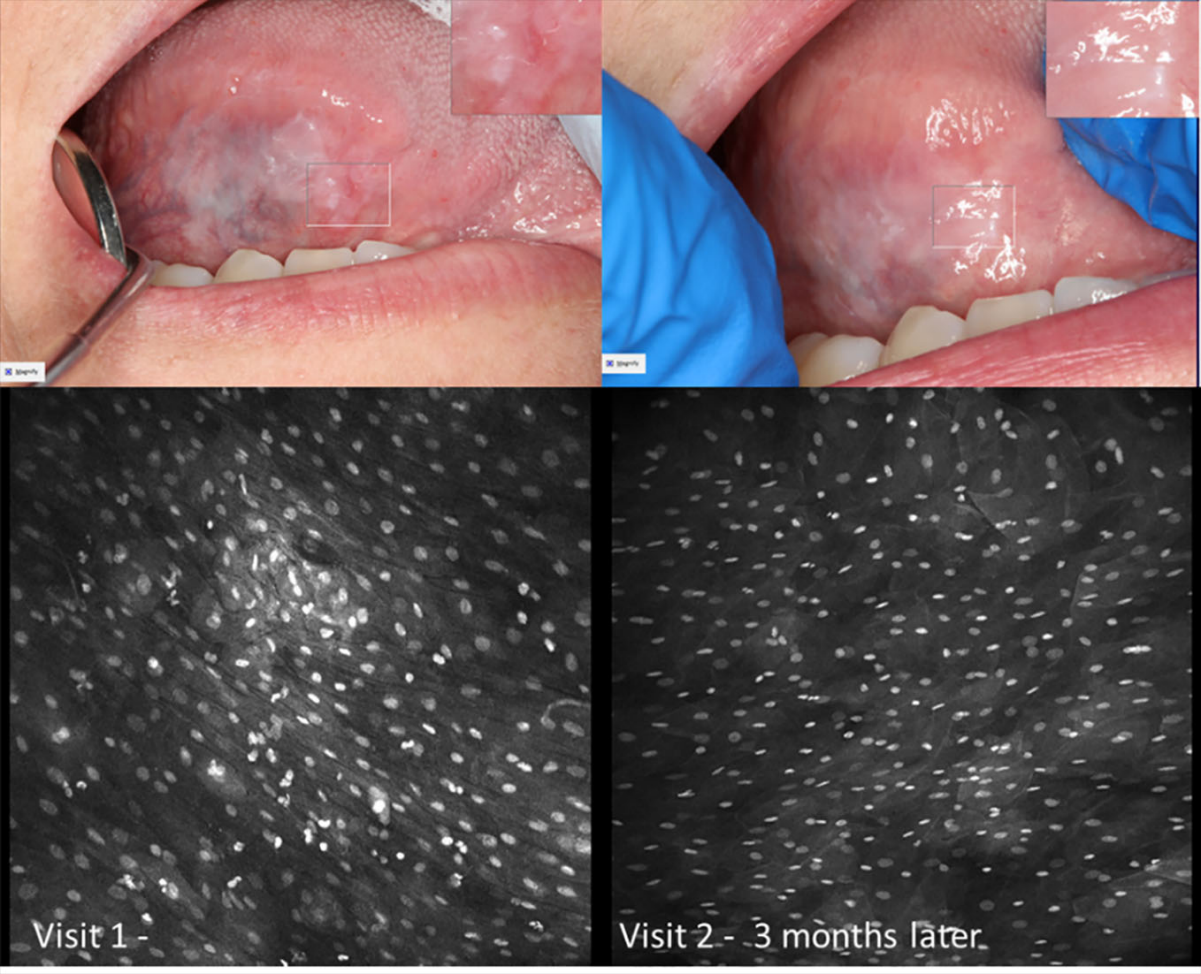
Macroscopic and micrographic images with topical 0.1% acriflavine demonstrating mild dysplasia over time. Macrographic photos, with right upper corner magnification viewed in MouthMap™, taken pre scalpel biopsy which was performed at Visit 1. Micrographically, the cellular outline and a regular intranuclear distance can be observed despite variability in nuclear size and fluorescence intensity. Images are 475µm (W) x 475µm (H).

**Figure 10:**
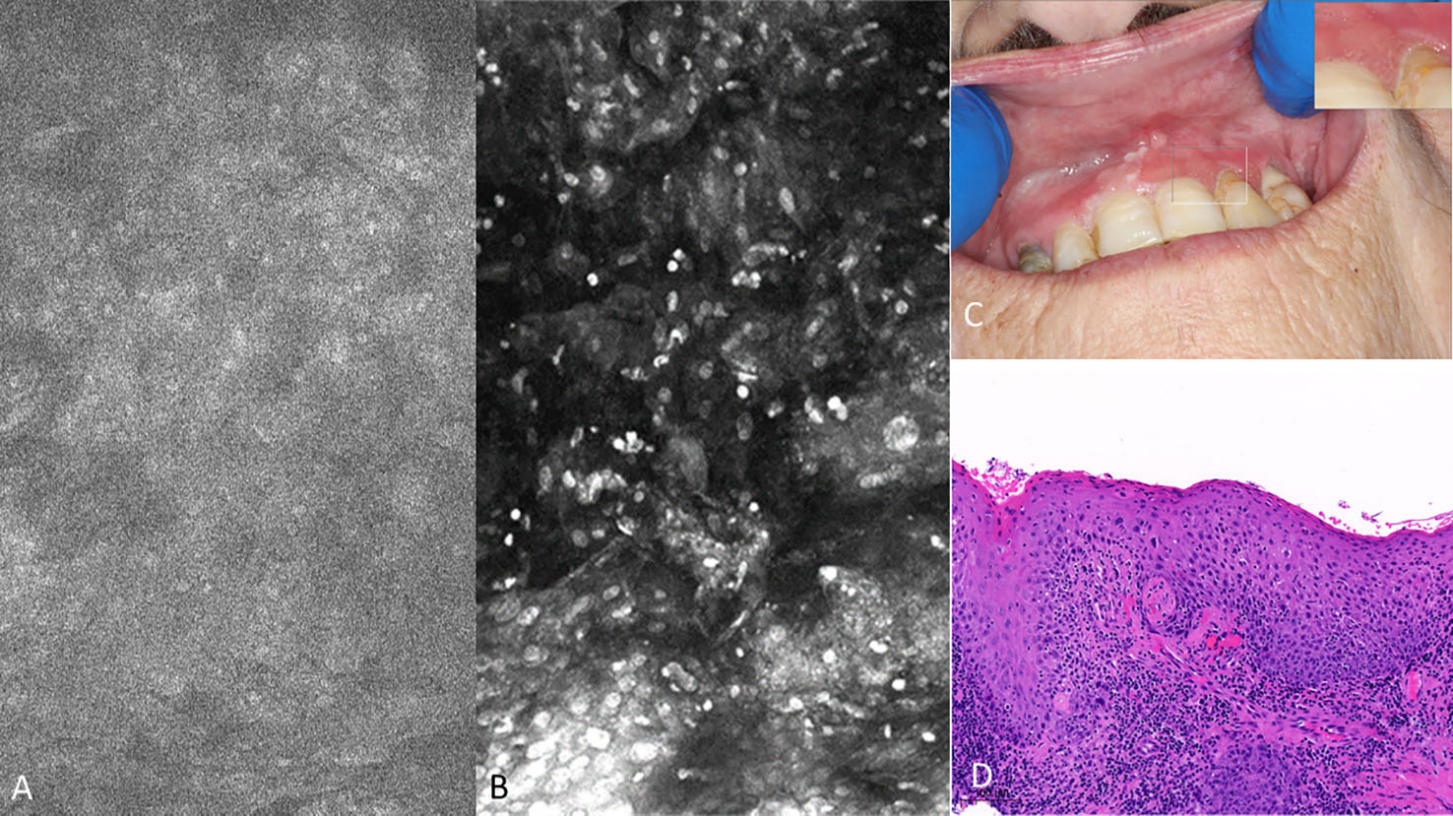
Mild dysplasia of the gingiva between the upper left central and lateral incisor **(A)** visualized with 1µM topical PARPi-FL demonstrating positive nuclei [275µm (W) x 475µm (H)] **(B)** visualized with topical 0.1% acriflavine [275µm (W) x 475µm (H)] **(C)** macrographic photo with right upper corner magnification views in MouthMap™ and **(D)** H&E histopathology (100 µm scale bar).

**Figure 11:**
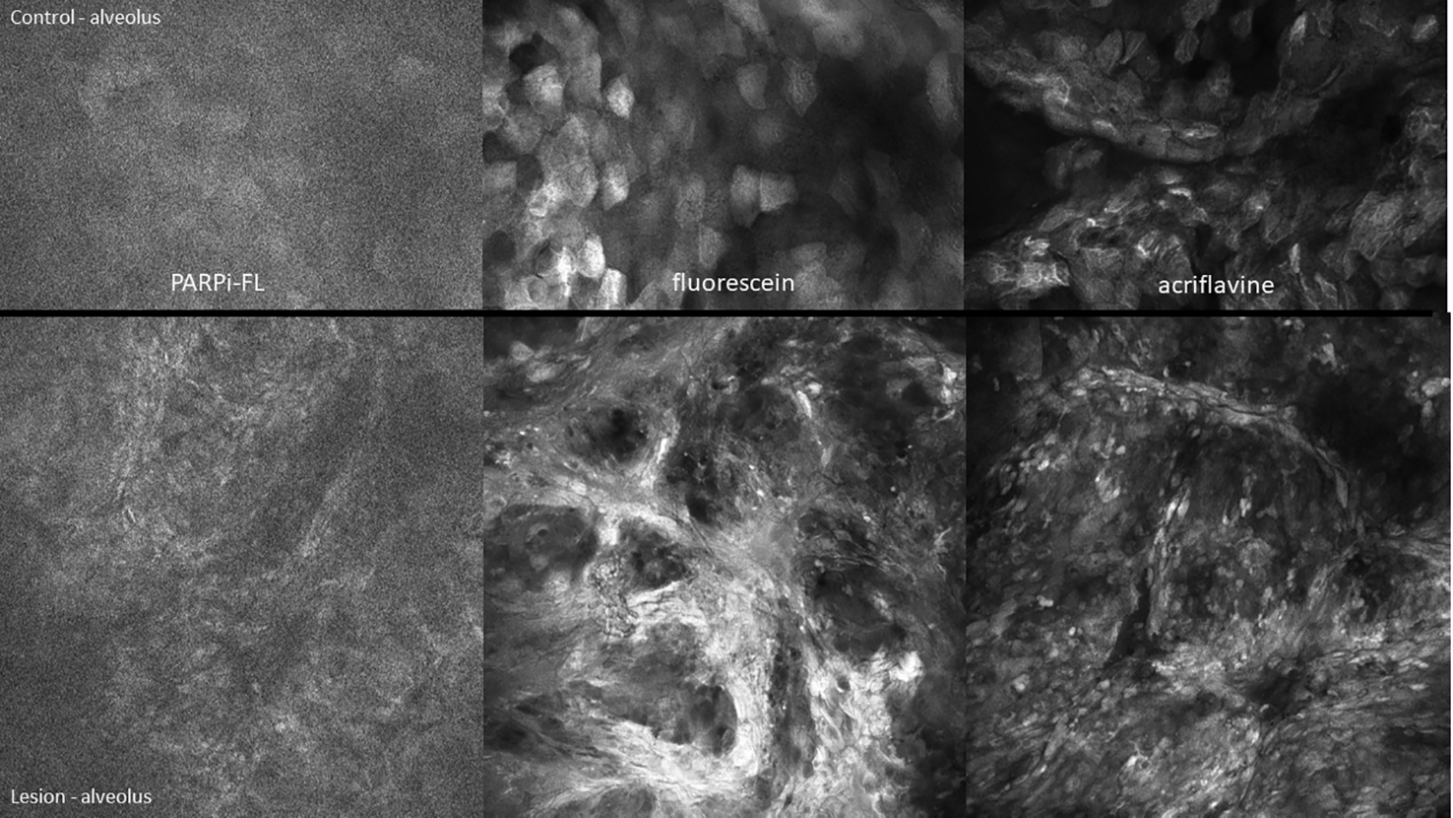
High-grade dysplasia of the lower left alveolus [lower panels 475µm (W) x 475µm (H)] and the contralateral clinically normal appearing mucosa [upper panels 475µm (W) x 300µm (H)]. Visualized using topical 1µM PARPi-FL, 0.1% fluorescein and 0.1% acriflavine.

Visual changes seen in low-grade oral dysplasia possess some shared changes with OSCC but are less overt. These include a subtle and progressive dishomogeneity in nuclear size, fluorescence, and inconsistent loss intranuclear distance. Cellular outline is often still visibly appreciated (**Figure 9**). Nonspecific PARPi-FL binding was seen in highly keratinized areas. PARPi-FL nuclear binding was also seen in low-grade dysplasia with demonstration of retained intranuclear distance (**Figures 10**, **11**). High grade dysplasia looked similar if not indistinguishable from OSCC (**Figure 11**).

### 3.6 Hyperkeratosis and hyperplasia

**Figure 12:**
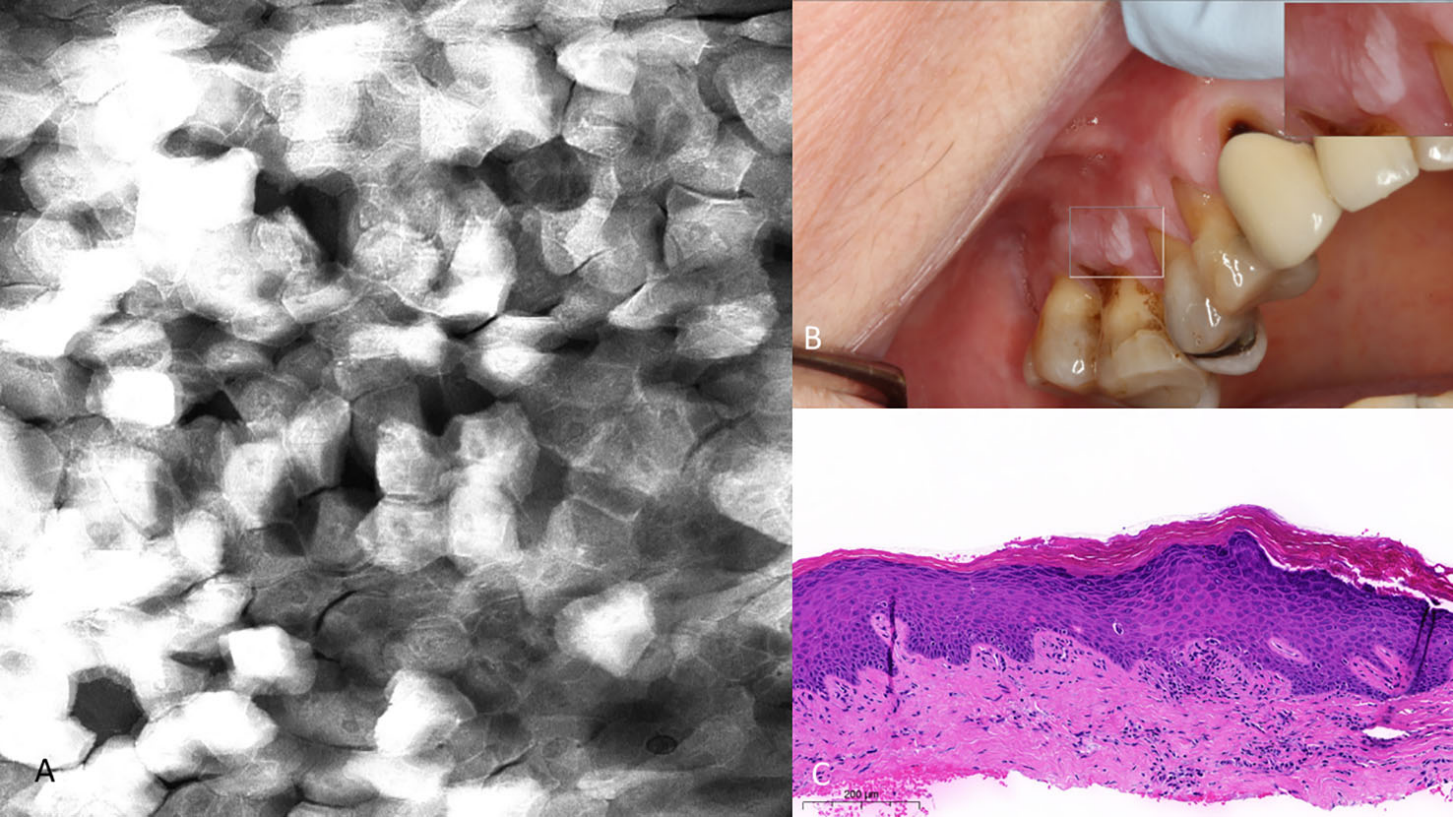
**(A)** Hyperkeratosis of the upper right gingiva (clinically non-dysplastic leukoplakia) with 0.1% topical fluorescein 475µm (W) x 475µm (H). Note the regularity of cellular dimensions despite variable fluorescent signal intensities. **(B)** Macrographic photo with right upper corner magnification views in MouthMap™. **(C)** H&E slide (scale bar 200µm).

**Figure 13:**
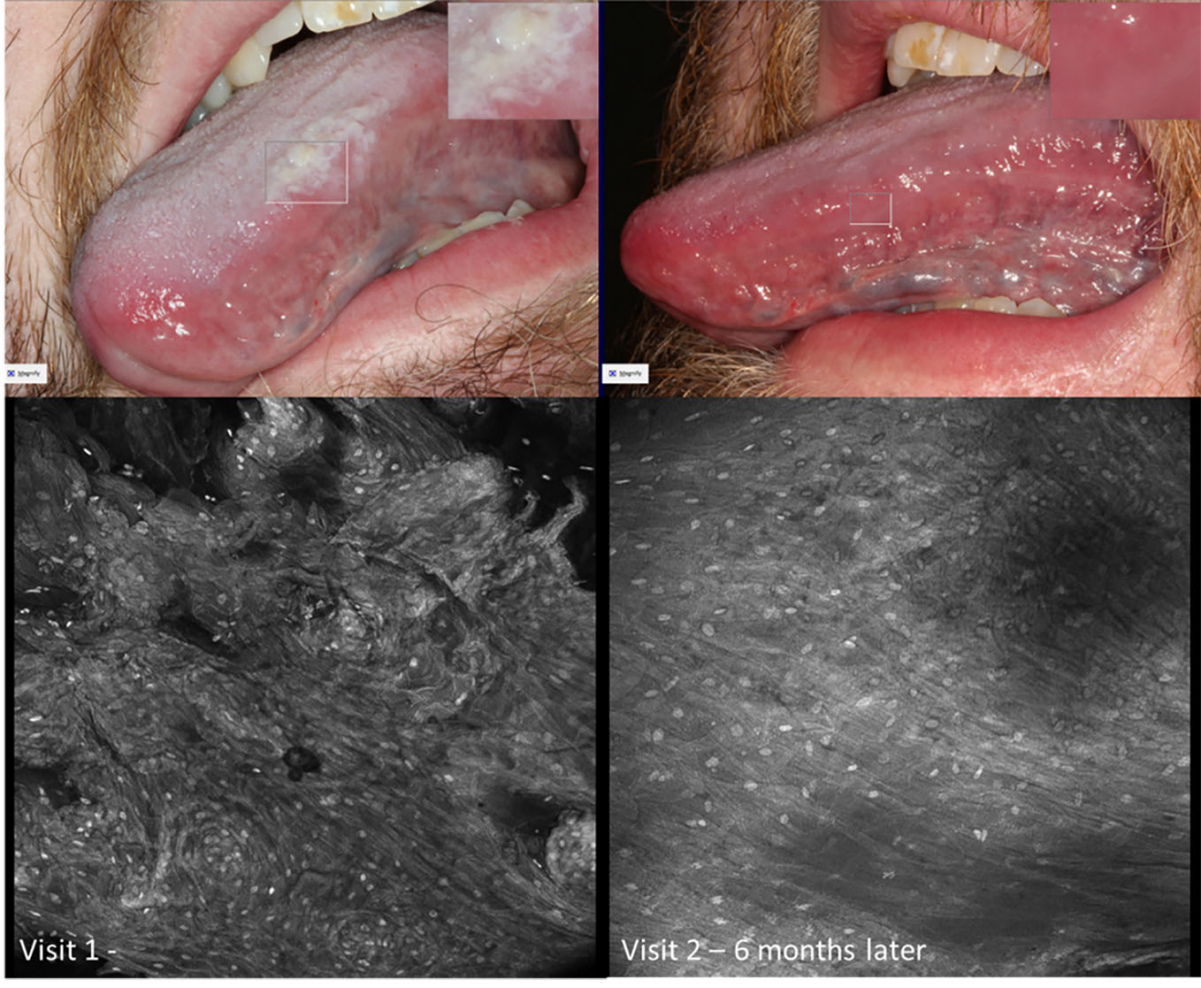
Micrographs of hyperkeratosis and hyperplasia observed with 0.1% fluorescein immediately before scalpel biopsy (performed at Visit 1) and corresponding macrographic photographs. Micrographs demonstrate consistent nuclei size and regular cellular outlines. The lesion resolved 6 months later showing clinically normal lateral tongue mucosa.

In the presence of hyperkeratosis, it was important to assess the cellular outline regularity as nuclei were not always observable (**Figure 12**). Secondly, it is imperative to note if there was a vertical component such as benign and malignant exophytic lesions because the confocal probe placement will invariably flatten the tissue, thereby influencing the presentation. Benign hyperkeratosis and hyperplasia could present with an appearance of pseudo-loss of internuclear distance, however the regular dimensions of both the cellular and nuclear profile remains intact (**Figure 13**).

### 3.7 Oral lichenoid inflammation

**Figure 14:**
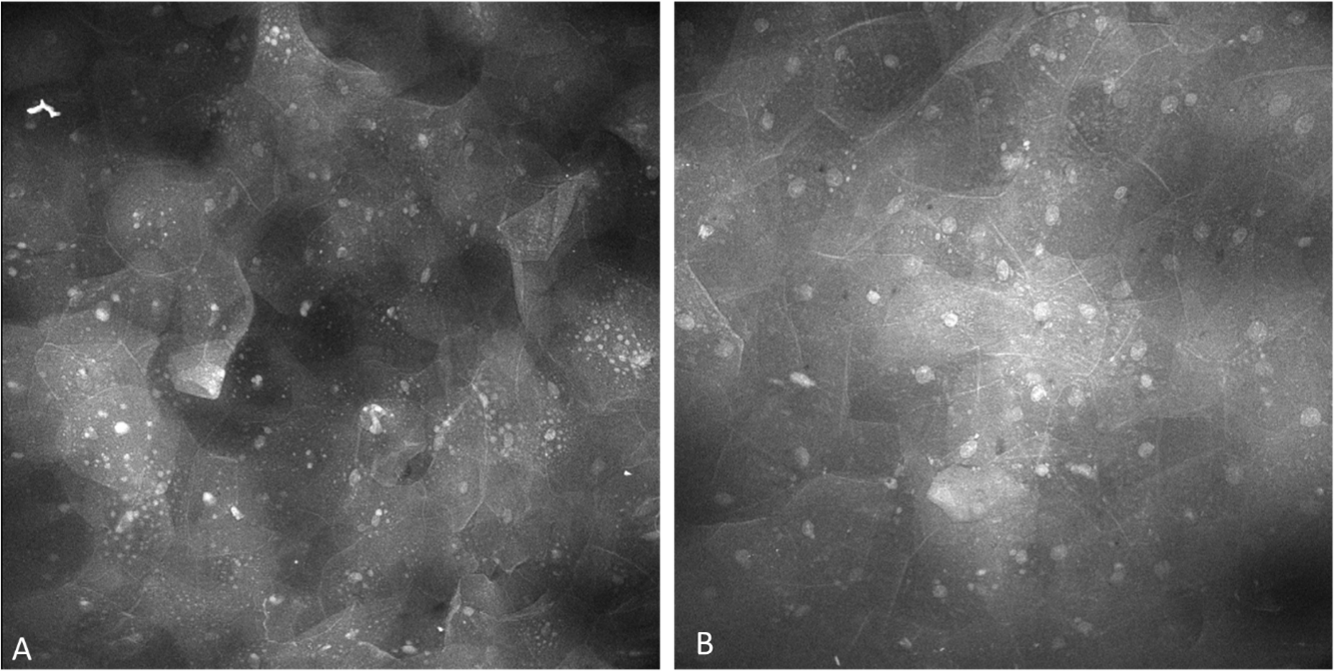
Perinuclear granules observed in lesions showing oral lichenoid inflammation, observed with both **(A)** topical 0.1% fluorescein and **(B)** topical 0.1% acriflavine.

The epithelium observed with oral lichenoid inflammation demonstrated regular nuclear size, fluorescence, spacing as well as cellular outline including with prominent and widespread perinuclear fluorescent granules (**Figure 14**). Perinuclear granular fluorescence was not infrequently observed in other diagnoses along with the features described above.

## 4 Discussion

Patients with OPMDs often have multiple lesions that can cover large areas within the oral cavity. Repeat biopsies of those lesions can cause a significant reduction in quality of life for the patient from both a functional and cosmetic perspective. High resolution *in vivo* microscopic assessment of the squamous epithelial oral mucosa allows simultaneous assessment of multiple lesions within the oral cavity. These micrograph images allow for serial comparison during subsequent confocal examinations, thus enabling analysis of tissue changes over time precluding the potential need for further biopsies.

In the present study we have performed digital biopsies of the mouth with a high resolution microscopic *in-vivo* visualization of the oral mucosa using 3 topical fluorescence agents using a protocol reproduced in over 300 imaging sets. We have demonstrated micrographs captured with the InVivage® scanned fibre device within an individual oral cavity over time. Our findings further advance understanding of the variation present in the *in vivo* microscopic landscape of abnormal oral mucosa in a novel way (31). Our findings build on work in the field which we recently summarized (32), including exploration with reflectance microscopy, fibre bundle, scanning mirror and precursor scanning fibre technology. Advancements in confocal microscopy proceed on the fronts of probe miniaturization, increased resolution and fluorescence targeting.

The proposed device and the feature of InVivage® System’s ability to detect only in-focus light provides a micro-anatomical cellular level that exceeds the resolution of currently available devices used for screening and surgical microscopes. We have demonstrated the utility of 3 fluorescent agents to acquire images with cellular level resolution.

One of the limitations of this workflow is that the agents are introduced topically. Whilst this is non-invasive and acceptable for the patient, the depth of penetration of the dyes determines the depth of visualization. For example, assessment could not be made regarding the connective tissue epithelium interdigitation or disruption or blood vessels within the connective tissue. This was the likely reason why severe dysplasia could not be differentiated from OSCC.

Exploration with PARPi-FL in visualizing OPMDs was important, but its application is likely of a higher clinical value in patients with a known diagnosis of OSCC where the extension of the lesion is the query. In patients with OSCC, the presence of known neoplasia allows determination of an accurate focal depth to survey the mucosa. Without this positive visualization reference, it is a challenge to determine where you are located within the mucosa to visualize non-neoplastic oral mucosa. This supports a call for the use of simultaneous 2-channel detection, and development and testing of new or modified fluorescent agents emitting light in different parts of the spectrum to enable both pancytoarchitectural visualisation of tissue structure and selective molecular targeted labelling of specific tissue features. Further categorical qualitative and quantitative analysis is required to definitively determine the strength in clinical information the 3 studied agents present. Further, the simultaneous application of acriflavine and fluorescein may be useful to explore.

Unlike physical pathology slides, digital biopsy images have very real potential for rapid comparison over time. Alongside the development of optimizing the digital biopsy image acquisition process, it is also crucial to have an annotation framework such as MouthMap™ which allows for easy annotation and reference to its corresponding intraoral location. In this way, digital health translation can bridge the laboratory to clinic spheres to improve and expedite chair side decision making.

This protocol offers real-time confocal microscopic imaging during oral mucosal assessment. Independent pattern recognition, qualitative as well as quantitative categorical assessments should be developed on captured image features. This should be in addition to focusing on visualizing features expected in traditional histopathology for the thorough assessment of oral mucosal abnormalities. Thereby, consensus based confocal based diagnostic criteria must be further developed and may be based on the consortium of previous confocal work in the field (10, 11)

There is also a need to establish imaging protocols as standard care so that acquisition and archiving of the device-captured images can be applied usefully in the clinical workflow. Digital capture obviously highlights the potential of augmentation for the application of machine learning and artificial intelligence. Although images captured by confocal microscopes have been candidates for deep learning (33), AI has yet to be applied to scanned fibre endomicroscopic *in vivo* captured images (32). In the future, these require further developments and a translation process back to chairside devices with the makings of a user-friendly workflow. These are the required steps to develop oral digital biopsies to be a crucial player in delivering the standard of care. As with any new technology, the practical clinical gains must be significant to justify any additional cost, training requirements and changes to workflow.

## 5 Conclusion

This study has demonstrated potential to increase diagnostic precision, augmenting current standard of care, by completing a more comprehensive imaging digital biopsy protocol of the patient without requiring more invasive scalpel biopsies. We have demonstrated successful implemented acquisition and annotation elements. In this way, the need for multiple biopsies may be reduced with onward facilitation of longitudinal comparison. High resolution microscopy of the oral cavity is demonstrated here with the clear capture of micrographic images of quality for pathological assessment of the oral cavity. We predict that the use of non-invasive high-resolution microscopy will have significant utility for the monitoring of oral potential malignant disorders, achieving not only the reduction in the need for scalpel biopsy whilst further allowing for increased data acquisition in less time, thus leading to higher diagnostic precision. This will result in a significant shift in the current paradigm of the visual and pathology-based assessment of individuals living with oral lesions and advance our ability to achieve earliest diagnosis, prevention and diminish mortality from oral cancer.

## Data availability statement

The original contributions presented in the study are included in the article/supplementary materials. Further inquiries can be directed to the corresponding author.

## Ethics statement

The studies involving human participants were reviewed and approved by University of Melbourne Medicine and Dentistry Human Ethics Sub-Committee (ID 1955205). The patients/participants provided their written informed consent to participate in this study.

## Author contributions

TY, LB and MM contributed to conception and design of the study. PD, TR and LB provided crucial study components for data capture. RR and CM provided organisation of the data capture. Data was acquired by IT, NB and TY. TY wrote the first draft of the manuscript. All authors contributed to manuscript revision, read, and approved the submitted version.
